# Biomineral Complex with Probiotic and Detoxifying Properties for Recovery After Radiotherapy

**DOI:** 10.3390/ijms27114794

**Published:** 2026-05-26

**Authors:** Olga Ilinskaya, Konstantin Vagin, William Kurdy, Galina Yakovleva, Nazira Karamova, Pavel Zelenikhin, Alexey Kolpakov, Yuri Zuev

**Affiliations:** 1 Department of Microbiology, Kazan (Volga-Region) Federal University, Kremlevskaya St., 18, Kazan 420008, Russia; william.m.kurdy@hotmail.com (W.K.); yakovleva_galina@mail.ru (G.Y.); nskaramova@mail.ru (N.K.); ljoscha@mail.ru (A.K.); 2 Federal Center for Toxicological, Radiation and Biological Safety, Science City-2, Kazan 420075, Russia; kostya9938@yandex.ru; 3 Kazan Institute of Biochemistry and Biophysics of the Kazan Scientific Center of the Russian Academy of Sciences, Lobachevsky St., 2, Kazan 420111, Russia; yufzuev@mail.ru

**Keywords:** radiotherapy, side effects, microbiome, chromosomal aberrations, clinoptilolite-containing carrier, *Lactobacillaceae*, alpha and beta diversity, functional analysis

## Abstract

Radiotherapy is a highly effective, safe cancer treatment, and about half of all cancer treatments involve lifesaving radiotherapy. Despite huge advances in technology that have made it safer and more effective, it is still not without side effects. They differ from patient to patient and can include fatigue, nausea, skin reactions, and hair loss, but dysbiosis is the most common complication associated with radiotherapy. Probiotics aimed at restoring the microbiome have found widespread use, but the problem of their rapid inactivation in the gastrointestinal tract has not yet been solved. Our study aims to confirm the effectiveness of a novel biomineral complex, based on a powdered clinoptilolite containing a rock loaded with lactobacilli for restoring the intestinal microbiome of mice exposed to radiation. Based on the 16S rRNA gene analysis, alpha-diversity and dynamics of changes in the fecal metagenome, as well as the functional potential of mice exposed to radiation, were studied, and the prospects of administering the biomineral complex to achieve positive effects were assessed. NMR analysis of the mineral carrier was carried out, and its safety was confirmed. Moreover, per os administration of the complex following irradiation led to a reduction in the level of chromosomal aberrations induced by irradiation. Thus, the biomineral complex has a microbiome-restoring effect and reduces radiation-induced clastogenesis.

## 1. Introduction

Radiotherapy (RT) is one of the primary methods in cancer management. About 30 to 50% of all cancer patients receive RT either alone or in combination with chemotherapy and surgery [1,2]. RT can cause side effects such as fatigue, depression, diarrhea, dysbiosis, sleep disorders, and so forth, impacting health-related quality of life. These side effects can be short- or long-term and vary among patients depending on the type of RT used. The composition and functional activity of the human microbiota play a crucial role in cancer pathogenesis, and the microbiota becomes dysregulated during tumorigenesis, as well as in response to RT. Advanced RT approaches such as the use of stereotactic body radiotherapy combined with microbiota modulation represent a promising strategy for sparing healthy tissue while maximizing immune-mediated antitumor effects [3].

Recent studies have revealed the gut microbiota’s active and crucial role not only in gastrointestinal cancers [4] but also in lung cancer patients with brain metastases. In these patients, key microorganisms associated with RT response were identified, and their potential as biomarkers was evaluated. The genera *Flavonifractor* and *Negativibacillus*, together with C-reactive protein and the systemic inflammation response index, were identified as independent predictors of radiotherapy response [5]. The identification of certain microorganisms in the microbiota can serve as a diagnostic marker for response to therapy. Moreover, not only for the intestinal microbiota but also for the respiratory microbiota of patients with advanced non-small cell lung cancer receiving PD-1/programmed death-ligand 1 blockade monotherapy, the objective response rate was higher in the high-diversity group compared with that of the low-diversity group [6]. Many modern studies confirm dysbiotic changes in the composition of the microbiome in cancer patients. Oral microbiome dysbiosis contributes to the development of oral cavity cancer [7]. Human colitis-associated colorectal carcinoma progression is accompanied by dysbiosis with enriched pathobionts. Microbiome alpha diversity in the nonneoplastic mucosa is positively correlated with colorectal cancer stage, and a high Shannon index predicts significantly shorter recurrence-free survival [4]. Colorectal laterally spreading tumors, early-stage lesions of colorectal cancer, are associated with gut microbiota dysbiosis [8]. Depletion of the bacterial microbiome induced by antibiotics leads to reduced tumor cell death following RT [9]. Thus, dysbiotic changes in the microbiome are characteristic of cancer; they persist throughout the course of cancer treatment and after its completion.

Probiotics, beneficial bacteria that can improve the balance of the gut microbiota, have shown promising potential in preventing cancer progression [10]. Several probiotic bacteria are currently used to restore the gut microbiota [11]. The lyophilized forms of the well-known probiotic bacteria *Lactobacillus* and *Bifidobacterium* are included in capsules that partially prevent their inactivation in the gastrointestinal tract (GIT) [12]. However, the release of lyophilized forms from capsules occurs almost immediately after entering the stomach [13]. During the 4–6 h spent in the stomach and small intestine, lyophilized bacteria do not have enough time to transform into vegetative forms [14]. Even some enteric capsules, which remain whole in the stomach and dissolve in the intestine, result in a massive emergence of vegetative forms from the lyophilizate only in the large intestine, which is densely populated by anaerobes, thereby making colonization of epithelia with probiotics difficult [15]. Therefore, the use of probiotic carriers that allow a gradual, prolonged release of bacteria is an important strategy for restoring the intestinal microbiota damaged during RT.

Nanosynergistic therapy is a novel therapeutic strategy that combines nanoparticles and probiotics or other agents to modulate the gut microbiota, increase drug bioavailability and stability, and reduce side effects. Dextran nanoparticles loaded with irinotecan, xylan-stearic acid conjugates loaded with capecitabine, and silver and gold nanoparticles have been shown to target specific gut pathogens to inhibit harmful microorganisms while promoting the growth of beneficial probiotics [4]. Zeolitic imidazolate frameworks (ZIFs), a subclass of metal-organic frameworks in which tetrahedrally coordinated transition-metal ions (e.g., Fe, Co, Zn) are connected by imidazolate linkers, are a platform for drug delivery and controlled drug release [16]. ZIF-8, which encapsulates mitoxantrone—an immunogenic cell death-inducing chemotherapeutic agent (MIT@ZIF-8)—was applied as a dual-functional nanomaterial for chemo-immunotherapy: a carrier to enhance tumor uptake of mitoxantrone for improved chemotherapy efficacy, and a pyroptosis inducer to amplify mitoxantrone-induced immunogenic cell death for augmented antitumor immune responses [17]. Different modifications of ZIFs are used to combat implant-associated infections [18] and serve as antibacterial platforms for the regeneration of infected skin wounds. [19]. ZIFs are topologically isomorphic to zeolites. The porous network of ZIFs consists of a central cavity (size ∼1.16 nm) connected to narrow apertures (size ∼0.34 nm) [20]. However, this pore size does not allow bacteria to be adsorbed. Therefore, natural minerals, in particular zeolites, are receiving increased attention for the delivery of probiotic bacteria into the GIT.

Clinoptilolite is a naturally occurring zeolite composed of microporous arrangements of silica and alumina tetrahedra, linked through shared oxygen atoms. It possesses a negatively charged open-framework porous structure, where the negative charge neutralization is achieved through cations (Na^+^, K^+^, Mg^2+^, Ca^2+^) capable of ion exchange. Compared to fibrous and lamellar zeolites, spherical clinoptilolite is less toxic [21,22,23] and is therefore used as a sorbent in agriculture, veterinary, and human medicine for contaminant removal and detoxification [24,25]. Clinoptilolite is not absorbed and is excreted with feces. There is no evidence that clinoptilolite is degraded during its passage through the gastrointestinal tract of animals [26].

Here, we investigated the effectiveness of a biomineral complex based on an active probiotic lactobacilli strain adsorbed on a mineral carrier—clinoptilolite-containing rock—in correcting the intestinal microbiota of experimental animals subjected to radiotherapy. The design of the preparation, the structure, porosity, and cytotoxicity of the carrier to eukaryotic cells, as well as important characteristics of the immobilized bacteria, such as survival, production of organic acids during long-term preservation, and resistance to gastrointestinal tract fluids, were described earlier [27]. In this study, the following objectives were addressed: (i) to investigate the gut microbiota of laboratory mice using 16S rRNA gene sequencing; (ii) to determine the alpha and beta diversity of the microbiota of non-treated and irradiated mice, and mice that received restorative therapy with the biomineral complex after irradiation; (iii) to identify the main groups of bacteria that changed quantitatively after irradiation and restorative therapy; and (iv) to analyze the frequency of chromosomal aberrations in the peripheral blood of mice before irradiation, after irraditation, and after restorative therapy. Finally, the main aim was to confirm the novel biomaterial’s potential for improving health status after RT.

## 2. Results

### 2.1. Frequency of Chromosomal Aberrations After Irradiation and Biomineral Complex Administration

The lifespan of red blood cells in mice is significantly shorter than that in humans, and averages 20–40 days. To capture chromosomal aberrations induced by radiation, we chose a time point of 12 days, approximately half the time required for red blood cell turnover. As a positive control, we analyzed the blood of mice given colchicine, a known inducer of chromosomal aberrations [28]. It was found that after 24 h of colchicine administration, the number of micronucleated erythrocytes increased by 2.6-fold compared to control mice (Table 1), confirming the validity of the test. Administration of the mineral carrier or the biomineral complex did not significantly alter the level of micronucleated erythrocytes. However, a single irradiation session, even after 12 days, increased the number of micronucleated erythrocytes by 1.6-fold. The administration of the mineral carrier or the biomineral complex following irradiation led to a significant reduction in the level of chromosomal aberrations in comparison to mice receiving only irradiation. The biomineral complex, lactobacilli immobilized on a carrier, was especially effective (Table 1).

### 2.2. Alpha Diversity of Mice Fecal Metagenome

Analysis of alpha diversity in the microbial communities of mice fecal samples revealed noticeable variations in the number of taxa (taxonomic richness index), the Shannon–Wiener, Simpson diversity, and evenness indices (Table 2). The highest number of OTUs was observed in the irradiated group with no follow-up treatment. The number of OTUs in this group on day 6 was 3486 and was 4434 on day 12 values that were 1.5- and 1.9-fold higher, respectively, than the mean across all other groups (2288 ± 420 OTUs). The same group showed the highest overall diversity coupled with the highest evenness (Shannon–Wiener index = 5.584 and evenness = 0.06005 on day 12).

### 2.3. Beta-Diversity of Mice Fecal Metagenomic Samples

**Figure 1:**
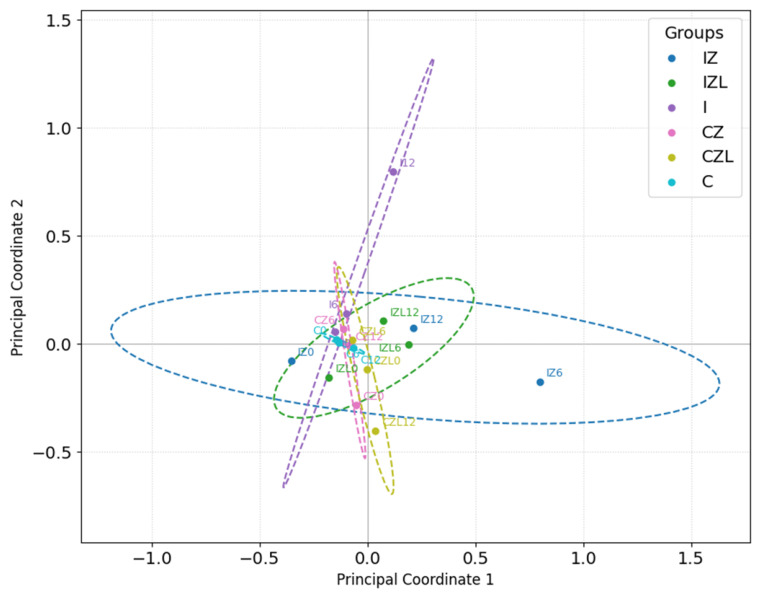
Beta-diversity and spatial distribution of six groups in the experiment.

Between-group divergence relative to the control group, intragroup stability, and temporal dynamics were visualized using the principal coordinate analysis (PCoA) based on Bray–Curtis index values (Figure 1). Analysis of the distances between group centroids revealed different treatment effects. The greatest similarity to the control group was observed in the CZ group, where d = 0.0817. On the other hand, the IZ and I groups showed the greatest divergence from the control group, where d = 0.3395 and d = 0.3363, respectively. These values indicate a major change in the bacterial communities. Intermediate divergence values were observed in the IZL group, where d = 0.1431, and the CZL group, where d = 0.1981.

Examining the homogeneity of intra-group variability revealed a unique time dynamic. Group C demonstrated very high intra-group temporal stability (distance range: 0.0188–0.0530), providing a baseline for comparison with all other groups. Converging trajectories (CZ and IZL) showed a trend toward intra-group stability. For example, the CZ distance from the centroid decreased from 0.2125 on day 0 to 0.0679 on day 12, implying a shift toward homogeneity. Diverging trajectories (CZL and I) demonstrated a trend toward intra-group instability. The CZL group showed a progressive trend: from 0.0504 on day 0 to 0.2401 on day 12. The IZ group showed high-intensity dynamics and the greatest initial instability, with the largest intra-group distances from the centroid on day 0 (0.5727) and on day 6 (0.5910). However, the distance to the centroid decreased sharply on day 12, probably indicating recovery.

### 2.4. Changes in the Structure of the Mice Fecal Metagenome After Irradiation and Preparation Administration

**Figure 2:**
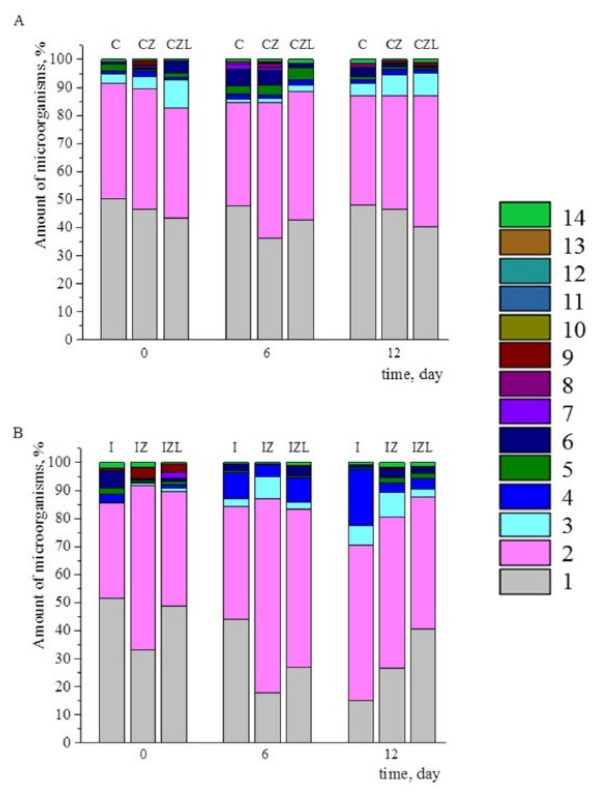
Metagenomic analysis of mouse feces at the phylum level: (**A**)—non-irradiated mice group; (**B**)—irradiated mice (dose 4 Gy); 1—*Bacteroidota*; 2—*Bacillota*; 3—*Actinomycetota*; 4—*Pseudomonadota*; 5—*Thermodesulfobacteriota*; 6—*Campylobacterota*; 7—*Cyanobacteriota*; 8—*Deferribacterota*; 9—*Verrucomicrobiota*; 10—*Chloroflexota*; 11—*Dependentiae*; 12—*Rhodothermota*; 13—*Spirochaetota*; 14—Other.

The metagenome of non-irradiated mice showed little change over the course of the experiment. A slight increase in the *Actinomycetota* phylum was noted toward the end of the experiment in mice receiving the mineral carrier or the biomineral complex (Figure 2A). However, the effect of radiation increased over time, manifesting as an increase in the relative abundance of *Bacillota* and *Pseudomonadota* and a decrease in the relative abundance of *Bacteroidota* (Figure 2B). In mice receiving the mineral carrier after irradiation, the destabilizing effect of radiation remained unchanged. Administration of the biomineral complex brought the microbiome composition closer to that of the control group by the end of the experiment (Figure 2).

**Figure 3:**
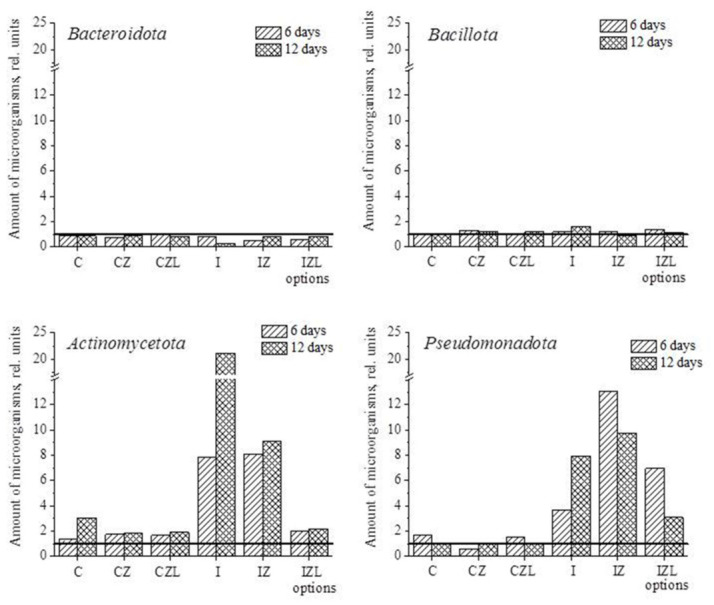
Changes in the relative abundances of major phyla in mouse feces after irradiation. One relative unit is the abundance on day 0 for each group.

The standard representation of phyla in the mouse fecal metagenome underemphasizes phyla with low relative abundance (Figure 2). However, changes in the microbiome are particularly revealing when relative abundances on day 0 are taken as a reference, and changes caused by irradiation and treatment are calculated relative to the initial phyla abundance values. Figure 3 shows that irradiation caused significant changes in the abundance of *Actinomycetota* and *Pseudomonadota*, while the administration of the biomineral complex brought these values closer to those of the control group. The administration of the carrier showed a less pronounced restorative effect (Figure 3).

At the family level, irradiation had a strong impact on the relative abundance of different families. Among the representatives of the phylum *Bacteroidota*, the families *Muribaculaceae*, *Prevotellaceae*, and *Bacteroidaceae* predominated in all mice fecal samples. These families are constant components of the mammalian microbiota. On day 12 post-irradiation, the relative abundances of *Muribaculaceae* and *Prevotellaceae* were reduced in mouse fecal samples by 22.5-fold and 53.4-fold, respectively. The administration of the biomineral complex restored the abundance of these families (Figure S1).

**Figure 4:**
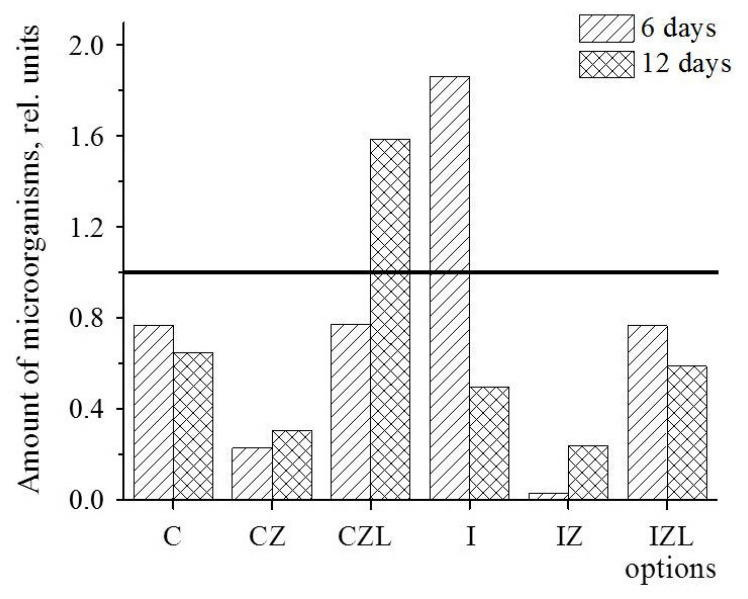
Changes in the relative abundance of *Lactobacillaceae* family in mouse fecal samples exposed to irradiation. One relative unit is the abundance on day 0 for each group.

Among the representatives of the phylum *Bacillota*, two families predominated in all mice fecal samples, *Lactobacillaceae* and *Lachnospiraceae* (Figure S2). We paid special attention to the abundance of lactobacilli in the samples. Irradiation resulted in a 1.9-fold increase in *Lactobacillaceae* in mouse fecal samples on day 6 of the experiment, followed by a decrease by day 12 (Figure 4). Compared to the irradiated-only group (I), the biomineral complex group (IZL) showed a 37.3-fold and 4.2-fold lower fecal abundance of lactobacilli on days 6 and 12, respectively. This reduced fecal shedding—combined with the IZL group’s convergence toward control-level relative abundance in Figure 4—supports the hypothesis that the complex enhances mucosal adhesion of administered lactobacilli.

Among the representatives of the phylum *Pseudomonadota*, two families predominated in all mice fecal samples, *Sutterellaceae* and *Enterobacteriaceae* (Figure S3). Irradiation of mice resulted in a 2.1-fold decrease in the abundance of the *Sutterellaceae* family on day 12 of the experiment, while the administration of either the carrier or the preparation of both drugs increased their abundance by an average of 1.8 ± 0.3-fold. A similar pattern was observed for members of the *Enterobacteriaceae* family: a 5.6-fold decrease after irradiation and 3.2- and 1.6-fold increases after the administration of the carrier or the preparation, respectively. Based on the data obtained, it is possible to identify a positive trend in the influence of the biomineral complex on the representation of beneficial bacteria in the intestines of mice exposed to irradiation.

### 2.5. Functional Analysis of Mouse Fecal Metagenome

**Figure 5:**
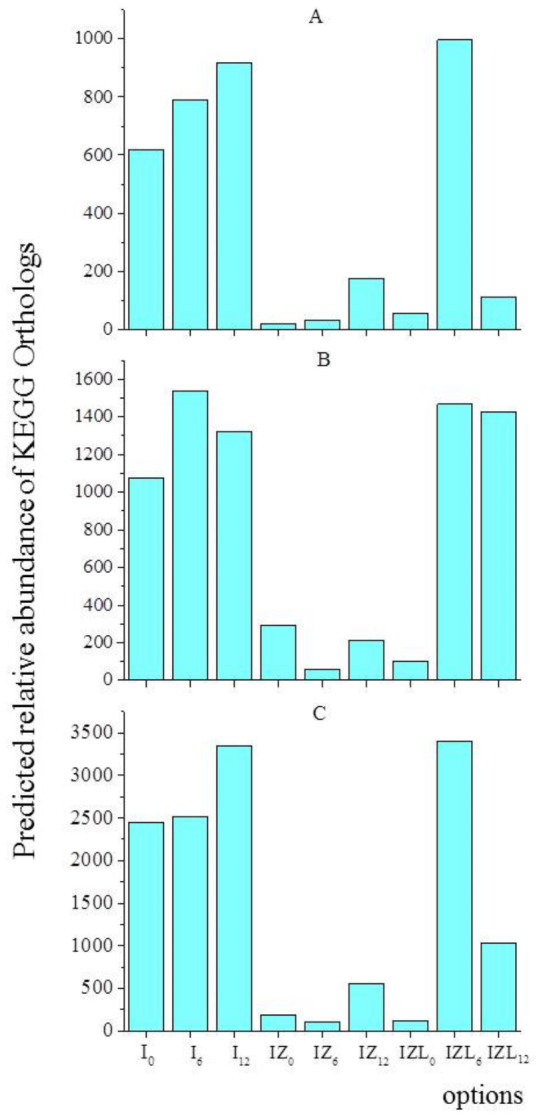
The number of genes in mouse fecal samples after irradiation. (**A**)—lipopolysaccharide biosynthesis, (**B**)—nucleotide excision repair, (**C**)—glutathione metabolism. *X*-axis labels: the numbers represent sampling days, I—irradiation without preparation intake, IZ—mineral carrier intake, IZL—biomineral complex intake.

We performed a functional bioinformatics analysis of predicted bacterial metabolic pathways (Supplementary Table S1), identifying pathways counteracting radiation damage compared with one of the main metabolic pathways–lipopolysaccharide biosynthesis (Figure 5A). Patterns of genes responsible for nucleotide excision repair (Figure 5B) and glutathione metabolism (Figure 5C) are similar to those for lipopolysaccharide biosynthesis.

Compared to non-irradiated controls (Table S1), irradiated mice showed a time-dependent increase in fecal abundance of bacteria harboring genes involved in nucleotide excision repair, glutathione metabolism, and lipopolysaccharide biosynthesis (Figure 5), consistent with radiation-induced epithelial disruption and enhanced bacterial shedding.

Mineral carrier administration after irradiation reduced the abundance of the studied genes in mouse fecal samples; biomineral complex administration increased bacterial elimination from the intestines on day 6 and decreased it again on day 12. Presumably, the introduction of lactobacilli on day 6 hindered gut bacterial adsorption, and by day 12, some of them remained in the intestines, which indirectly indicates the restored epithelial adsorption of bacteria. This pattern can be traced in both the main phyla (Figure 3) and the family *Lactobacillaceae* (Figure 4). Dynamic changes in gene counts are similar for all metabolic pathways (Supplementary Table S1, Figure 5). These data show that there is a direct correlation between the number of OTUs and the number of functional genes.

### 2.6. NMR Spectrum of the Aqueous Extract of the Mineral Carrier

**Figure 6:**
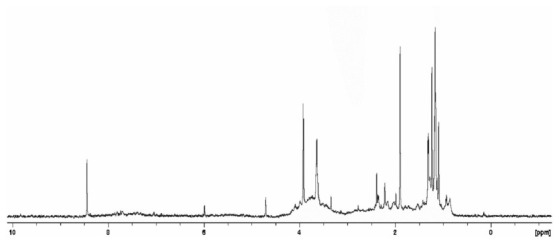
^1^H NMR spectrum of a zeolite sample in D_2_O.

Figure 6 shows the nuclear magnetic resonance (NMR) spectrum of the aqueous extract of the carrier used for probiotic bacteria immobilization. The proton signals in the spectrum are low-intensity, indicating a low concentration of dissolved molecules in the solution. Relatively intense narrow signals in the 1–1.5 ppm region are related to the CH_3_ groups of low-molecular-weight compounds. The presence of low-intensity signals in the 2–4 ppm and 7–8 ppm regions may indicate the presence of a small amount of organic macromolecules in the sample, such as proteins and nucleic acids. In general, it is possible to conclude that the carrier sample contains only trace quantities of water-soluble substances. These data once again confirm the chemical inertness of the carrier and the absence of significant soluble organic impurities. The biological safety and non-toxicity of the carrier were previously established through in vitro cytotoxicity and in vivo evaluations [27].

## 3. Discussion

Gastrointestinal toxicity, including radiation enteritis and mental fatigue, is a common side effect of radiotherapy that substantially reduces patients’ quality of life. Among the various organs affected, the gut is particularly sensitive to radiation-induced damage [29]. Currently, there are no effective drugs available for the prevention or treatment of radiation-induced enteropathy or radiation-induced brain injury, both of which impair cognitive function, leading to severe complications or even death. Numerous current studies have confirmed that intestinal microbiota dysfunction is an important factor in the formation of these diseases, which are major adverse events following radiotherapy of malignant tumors. Irradiation causes changes in the composition of the flora and a decrease in its diversity, which is mainly manifested by a decline in beneficial bacterial species such as *Lactobacilli* and *Bifidobacteria* [30,31,32,33].

It is now established that probiotic interventions can protect the rectal mucosa by reducing inflammation and modulating the mucosa-associated microbiota. Preventive effects of combined live *Bifidobacterium*, *Lactobacillus*, *Enterococcus*, and *Bacillus cereus* tablets were shown in patients with radiation pneumonitis [34]. Probiotics, including *Bacillus licheniformis*, have been shown to mitigate intestinal inflammation and mucositis by modulating gut microbiota and immune responses. *Bacillus licheniformis* supplementation effectively alleviates craniospinal irradiation-induced gastrointestinal dysfunction and inflammation in pediatric patients with medulloblastoma, but does not significantly improve their survival rates [35]. Oral administration of *Lactobacillus reuteri*, which releases interleukin-22 at 24 h after total-body irradiation, mitigates damage to the intestine. Furthermore, this probiotic facilitated whole-abdomen irradiation when added to paclitaxel and carboplatin chemotherapy, thereby further increasing survival [36]. Probiotics effectively reduced the incidence of CyberKnife-associated radiation pneumonitis among patients with pulmonary malignancies, delayed radiation pneumonitis onset, and improved their quality of life [34]. The effect of probiotics extends not only to improving the gut microbiome but also to maintaining normal brain function. Patients taking *Lactobacillus* probiotics had better role functioning, emotional balance, and cognitive clarity after six weeks; probiotics helped patients adapt better to treatment stress and protect the gut–brain axis during radiotherapy [37]. Bacterial metabolites also have positive effects on human health damaged after irradiation. Indole-3-carboxaldehyde, derived from the intestinal microbiota, enhanced the abundance of probiotics, activated the AhR/IL-10/Wnt signaling pathway to promote intestinal epithelial proliferation, and demonstrated potential clinical application value for the treatment of radiation-induced damage [38].

Radiation-induced damage to the villi height and mucosal thickness was significantly mitigated by probiotic treatment (*p* < 0.01). Probiotics reduced radiation-induced numbers of pyknotic cells and neuronal inflammation in the cortex (*p* < 0.01). Altogether, probiotic treatment helped mitigate radiation-induced intestinal and neuronal damage [39]. Meta-analyses of 16 randomized controlled trials including 2097 patients have established that compared with the placebo groups, oral probiotics significantly reduced the side effects caused by radiotherapy and chemotherapy in various types of cancer, such as head and neck cancer, pelvic and abdominal cancer, breast cancer, lung cancer, etc. [40].

Thus, sufficient evidence has been obtained on the benefits of probiotics for the recovery of patients after radiotherapy. A modern understanding of the functional role of the gut microbiota in the body’s immunological, metabolic, and neurological status has prompted the development of new probiotic formulations incorporating a sorbent carrier or a polymer capsule to protect bacteria from the harsh gastric environment. Encapsulation technologies, particularly those using natural biopolymers such as alginate, chitosan, pectin, carrageenan, and gelatin, have significantly improved probiotic viability, shelf stability, and targeted release. Ensuring prolonged probiotic release in the gastrointestinal tract remains a serious and pressing challenge that cannot be addressed without accumulating fundamental knowledge about the carrier structure and its dynamic interactions with the target agent, as well as demonstrable data on the stability, efficacy, and safety of the formulation. Various technologies are used to ensure the stability of probiotics in gastric juice. *Bacillus cereus,* possessing antioxidant ability and covered with a polydopamine/chitosan layer-by-layer assembly, exhibited tolerance to ionizing radiation, freeze drying, long-term preservation at room temperature, and gastric acid [41]. We have developed a new *Lactobacillus* preparation immobilized on a mineral carrier. The clinoptilolite-containing carrier has been shown to be non-toxic [27] and contains only trace quantities of water-soluble substances (Figure 6), rendering it essentially inert. When loaded with lactobacilli, it provided prolonged stepwise release of bacteria over 12 h [27]. In this study, we demonstrated that this biomineral complex significantly reduced the frequency of chromosomal aberrations caused by radiation, restoring values to control levels (Table 1). Clinoptilolite was found to be the most effective adsorbent of cesium in vitro. When used in vivo, it significantly reduced the absorption of cesium by sheep fed contaminated herbage [42]. In veterinary medicine, zeolite improves the fitness of pets and removes radioactive elements, aflatoxins, and poisons. Zeolite also exhibits antioxidant, hemostatic, and antidiarrheal properties, projected for human care [43]. Moreover, in the Ames test, we have established that cell suspension and supernatant of *Lactobacillus* cultures possessed antimutagenic activity against sodium azide and 2-nitrofluorene [44], thereby further enhancing the detoxifying effect of the biomineral complex. The detoxifying properties are associated not only with clinoptilolite, but also with lactobacilli through direct binding of toxic compounds, influencing their biotransformation, and with the activity of secreted metabolites.

Alpha-diversity of mice fecal samples showed that the control group had very high intra-group temporal stability. The highest number of OTUs was observed in the irradiated group (Table 2), which demonstrated high-intensity dynamics and the greatest initial instability (Figure 1). Established irradiation-induced damage to the villi height and mucosal thickness [34] probably leads to disruption of intestinal microbiota adhesion and the release of many bacterial taxa into feces (Table 2). Beta-diversity assessment revealed a trend toward intra-group stability in the group of mice given the biomineral complex after irradiation (Figure 1). These data allow us to consider the effect of the complex to be positive. The influence of the complex on the abundance of *Actinomycetota* and *Pseudomonadota* can also be considered positive because significant changes in this parameter induced by irradiation shifted closer to the control group (Figure 2). It should be noted that on day 12 after irradiation, the ratio of *Bacteroidota*/*Bacillota* in the mouse fecal metagenome significantly decreased (Figure 2B), and administration of the complex restored it to the control level (Figure 2A). Overall, we found that families *Muribaculaceae*, *Prevotellaceae*, and *Bacteroidaceae*, which are constant components of the mammalian microbiota, predominated in all mouse fecal samples and significantly decreased on day 12 after irradiation (Figure 3). The administration of the biomineral complex restored the abundance of these families (Figure S1).

In similar experiments, Zhao et al. [45] found that during the recovery stage of acute radiation-induced intestinal injury, after the seventh day of radiation, the diversity of the mouse gut microbiota decreased overall, with the relative abundance of the phyla *Proteobacteria* and *Bacteroides* increasing. In our study, bacteria belonging to the phyla *Pseudomonadota* (previous name *Proteobacteria*) and *Bacteroidota* (previous name *Bacteroides*) also increased (Figure 3). Analysis of lactobacilli abundance in mouse feces revealed that on day 6 after irradiation, abundance increased, but on day 12, it decreased, which indicates the restoration of the normal intestinal metagenome (Figure 4). The biomineral complex reduced the abundance of lactobacilli by ~37-fold on day 6 and by ~4-fold on day 12, which indicates the adhesion of lactobacilli from the preparation to the mice’ intestinal epithelium. We adhere to the concept that the release of bacteria in feces indicates their elimination from the intestinal epithelium, which definitely indicates a disruption of the intestinal microbiome caused by irradiation. Thus, the restoration of the gut microbiome is associated with a reduction in the eliminated taxa in the feces of mice, which was shown in our experiments.

While pooled-sample metagenomics enabled robust detection of group-level microbiome trends, it precludes statistical evaluation of within-group variability. This design prioritizes detection of treatment-driven community shifts over assessment of inter-individual variability, consistent with exploratory microbiome study frameworks. Future studies with individual-level sequencing will be valuable to confirm these observations and assess inter-animal heterogeneity.

We focused on the functional bioinformatics analysis of metabolic pathways counteracting radiation damage, namely, glutathione metabolism, nucleotide excision repair, and biosynthesis of lipopolysaccharides as one of the main metabolic pathways (Figure 5). The patterns of dynamic changes in gene counts were similar for all metabolic pathways (Supplementary Table S1, Figure 5) and can be traced both in the main phyla (Figure 3) and in the *Lactobacillaceae* family (Figure 4), reflecting the direct relationship between the number of OTUs and the number of functional genes. The number of predicted gene copies in the feces of mice that did not receive the preparations increased after irradiation, while preparation administration reduced their number, indirectly indicating restored epithelial adsorption of gut bacteria and introduced lactobacilli.

Experimental data indicate that radiation exposure disrupts both the gut microbiota and metabolome during the acute injury phase, reducing beneficial bacteria such as *Ruminococcaceae* and *Bifidobacterium* [46]. Our results also indicated a high level of radiation-induced elimination of beneficial *Lactobacillaceae* from the intestines (Figure 4). However, we did not observe a significant difference between the yield of beneficial versus harmful bacteria. Radiation has been shown to damage not only the microbiome but also the intestinal epithelium. Intestinal organoids derived from the irradiated mice showed defects in budding and mucin expression, suggesting a detrimental effect of irradiation on the intestinal stemness and differentiation, whereas the indigenous gut bacterium *L. acidophilus* enhanced intestinal epithelial function [47]. Our data also confirm that the use of lactobacilli in a biomineral complex is justified for restoring the microbiome after radiotherapy. It is important to note that gut microbiota cannot be used as a sensitive biomarker at the prodromal stage in acute radiation-induced intestinal injury, but it is a potential biomarker at the critical stage, and interventions are needed to restore radiation-induced intestinal injury [45]. Thus, the administration of probiotics is an effective strategy to protect against irradiation and subsequent dysbiosis.

## 4. Materials and Methods

### 4.1. Animals

**Figure 7:**
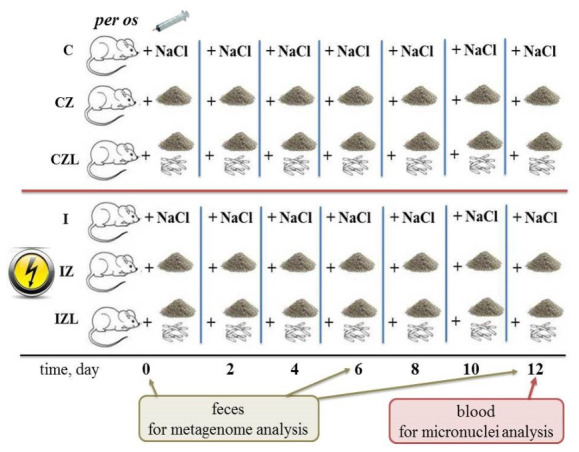
Scheme of experiments.

The experiments were performed on male white laboratory mice in accordance with the Directive of the Council of the European Communities (24.11. 86/609/EEC) and were approved by the Local Ethics Committee of Kazan Federal University (protocol No. 8 of 5 May 2015, protocol No. 33 of 25 November 2021). To simulate acute radiation sickness, animals were exposed to a single relatively uniform gamma irradiation in the biological gamma installation GUB-20, which contains sealed radionuclide sources of the IGI-Ts-9-1 type based on the radionuclide ^137^Cs, GUB-20 “Puma” (Russia, Saint Petersburg), a radiation source of ^137^Cs with an exposure dose rate of 1.99 × 10^−5^ A/kg (4.633 R/min) and with a dose field unevenness of 13.3%. The irradiation was delivered as a single dose of 4 Gy, sufficient to cause acute radiation sickness [45,48]. The animals were kept in standard vivarium conditions (air temperature 18–24 °C, relative air humidity 40–80%). Animals’ access to food and water was not limited (feeding regimen: *ad libitum*) according to the veterinary certificate of the Main Veterinary Directorate of the Republic of Tatarstan-216 No. 018687 dated 30 November 2021. Before the experiment, the animals were quarantined for 14 days, after which the mice were divided into 6 groups, with 5 individuals in each group: control mice without treatment (C); control mice receiving the mineral carrier (CZ); control mice receiving the biomineral complex (CZL); irradiated mice (I); irradiated mice receiving the mineral carrier (IZ) and irradiated mice receiving the biomineral complex (IZL). The mineral carrier (100 mg/mL of saline solution) and the biomineral complex (one ampule containing a 100 mg sample diluted in 1 mL of saline solution) were administered perorally at a dose of 0.2 mL per animal every two days for 12 days. The control group was administered a saline solution. The experimental scheme is shown in Figure 7.

### 4.2. Biomineral Complex

The clinoptilolite-containing rock from the Tatarsko-Shatrashanskoe zeolite deposit, Russia, was ground to microgranules smaller than 30 μm using an electric mill (Homaider, Guangzhou, China). High-temperature treatment of the samples was carried out for 30 min at a temperature of 500 °C (EKPS50 muffle furnace, model 5007, Smolensk, Russia). Thermal activation led to an increase in the porosity of the zeolite due to dehydration and combustion of residual organic matter, and is a process widely used in industry and agriculture [49]. Additionally, the zeolite samples were washed twice with 96% ethanol and dried at room temperature.

*Lactobacillus plantarum* B-11007 (new taxonomic name *Lactiplantibacillus plantarum*) registered in the VKPM collection, Russia, was isolated from commercial probiotic Lactobacterin dry (Biomed, Moscow, Russia) and cultivated in MRS medium (g/L: yeast extract—4.0, meat extract—10.0, casein hydrolyzate—10.0, glucose—20.0, ammonium citrate—2.0, sodium acetate—5.0, KH_2_PO_4_—2.0, MgSO_4_ × 7H_2_O—0.2, MnSO_4_ × 4H_2_O—0.05; pH 6.2–6.5) under microaerophilic conditions.

The Djuki’c–Vukovi’c technique, with some modifications, was used for immobilization [27,50]. A 16 h culture in a volume of 200 mL was subjected to centrifugation at 10,000 rpm for 5 min, and the sediment was washed twice with sterile 0.9% NaCl and resuspended in 200 mL of fresh MRS medium with the addition of clinoptilolite (5% of the total volume). The number of lactobacilli in the suspension averaged 5.8–6.0 × 10^8^ colony-forming units, CFU/mL. The suspension was incubated at 37 °C on a shaker (180 rpm, 20 h) and then centrifuged (1000 rpm, 5 min). The supernatant containing non-immobilized cells was removed; the sediment was washed twice with phosphate-buffered saline (0.01 M, pH 7.4) and used for lyophilization. The immobilization coefficient, reflecting the sorption capacity of the carrier, was calculated as the ratio between the number of viable cells (CFU/mL) in the suspension before immobilization and in the supernatant after immobilization, centrifugation, and washing twice with phosphate-buffered saline. The immobilization coefficient was expressed as a percentage, taking the initial number of lactobacilli in the suspension as 100%, and reached 96–98%. The bacterial pellet containing 100 mg of the clinoptilolite was suspended in 0.5 mL of lyoprotective sucrose–gelatin–milk medium (g/L: sucrose—100.0, gelatin—15.0, and skimmed milk powder—60.0) under aseptic conditions and packaged in glass ampoules using a polypropylene catheter. The necks of the ampoules were closed with sterile aluminum caps and frozen at −70 °C, after which they were subjected to dehydration for 5 h using a FreeZone Dryer (Labconco Corporation, Kansas City, MO, USA) at a vacuum depth of 0.035 torr.

### 4.3. Micronucleus Assay

Detection of micronuclei in normally anucleated erythrocytes is considered to be a consequence of the clastogenic and aneugenic effects of genotoxicants in vivo [51,52]. The count of micronuclei in mouse peripheral blood erythrocytes was performed 12 days after irradiation. As a negative control, animals were administered saline, and as a positive control, 0.125 mg/kg colchicine (Serva, Heidelberg, Germany). Colchicine is an aneugen whose main genotoxic effect is the induction of chromosomal segregation errors and aneuploidy by disrupting microtubule assembly. It induces micronuclei in the peripheral blood at 0.125–2 mg/kg [53]; therefore, the dose of 0.125 mg/kg was chosen for our experiments. Blood smears from the tail vein of mice were fixed with methanol, stained with Giemsa dye (Koch-Light Laboratories, Berkshire, England), and the number of micronuclei per 1000–2000 erythrocytes was counted in each animal group.

### 4.4. Metagenome Analysis

Molecular genetic analysis of the microbial community was performed according to the following protocol: mixed mouse fecal samples from each group were homogenized, and ~1 g of each sample was sterilely aspirated for DNA extraction using a Miniprep Kit (Axygen, Union City, CA, USA) according to the manufacturer’s protocol. Thus, metagenomic data represent composite group profiles (n = 1 per group/timepoint). This design prioritizes detection of treatment-driven community shifts over assessment of inter-individual variability, consistent with exploratory microbiome study frameworks. DNA concentration was determined and normalized to ≤10 ng/µL. The quality of DNA extraction was assessed by confirming amplification of the 16S rRNA gene targeting the V3–V4 region of the bacterial 16S rRNA gene using PCR and gel electrophoresis. Library preparation and sequencing were performed using the Illumina MiSeq. Preprocessing and subsequent sequence analysis were performed using the Mothur software package v.1.48.0 [54] on the Galaxy platform [55]. Data analysis was performed according to the Galaxy Training manual [56]. Operational taxonomic units (OTUs) were selected at a 97% identity threshold and classified using the SILVA 138.2 reference dataset [57].

### 4.5. Biodiversity and Functional Analysis

Alpha diversity indices, namely observed richness, Shannon, and Simpson, and beta-diversity Bray–Curtis and Dice–Sorensen similarities matrices were calculated using the PAST v4.02 software package. Principal coordinate analysis (PCoA) was used to illustrate the beta diversity and spatial distribution of all six groups in the experiment: C, CZ, CZL, I, IZ, and IZL. To assess group stability and treatment effects, 95% confidence ellipses were calculated, and Euclidean distances were determined to measure intra-group variance and between-group variance. PCoA ordination based on the Bray–Curtis dissimilarity matrix was performed and visualized in RStudio version 4.4.3 using the vegan and ggplot2 packages.

Functional prediction of bacterial communities’ pathways was carried out using PICRUSt2 [58] via the Galaxy platform (usegalaxy.org). The ASV table and representative sequences were processed with the default PICRUSt2 workflow to predict KEGG Ortholog abundances based on phylogenetic placement.

### 4.6. ^1^H NMR Spectroscopy

The mineral carrier (0.5 g) was suspended in 1.5 mL of deuterated water for 24 h. The supernatant was taken to fill the NMR ampule for further spectral measurements. NMR spectra were obtained on the Bruker Avance III 600 MHz spectrometer (Billerica, MA, USA) equipped with a triple-resonance TBI probe, z-gradients, and the BCU05 temperature control unit. The spectra were recorded at a temperature of 30 °C. One-dimensional proton spectra of the water system were recorded using a standard water-suppressed pulse sequence (zgesgp, rg = 2050, ns = 1024). The Topspin software 5.0 release was used for data processing.

### 4.7. Statistics

Data processing and plotting of taxa distribution among samples were performed using the standard Excel 7.0 software. To assess differences between mice blood samples when analyzing the results of the micronucleus test, the nonparametric Mann–Whitney criterion (U-test) was used, according to which U < 1 is the significance zone, U = 1–4 is the uncertainty zone, and U ≥ 4 is the zone of insignificance of differences.

## 5. Conclusions

Because of probiotics’ rapid inactivation in the gastrointestinal tract, the problem of restoring the intestinal microbiome after radiotherapy with beneficial bacteria remains relevant. Our results confirmed that the developed biomineral complex containing immobilized *Lactobacillaceae* on a mineral carrier, clinoptilolite-containing rock, restored irradiation-induced dysbiosis in mice thanks to its detoxifying properties and the gradual release of bacteria from the carrier, which gives them an enhanced likelihood of maintaining viability and adhering to intestinal epithelial cells. Irradiation caused significant changes in the abundance of *Actinomycetota* and *Pseudomonadota*, while the administration of the biomineral complex brought these values closer to those of the control group. Not only the restoration of the microbiome but also the elimination of clastogenic/aneugenic effects of radiation allow us to consider the biomineral complex as a promising drug for reducing side effects of radiotherapy.

## Figures and Tables

**Table 1:** Clastogenic/aneugenic effect of irradiation and treatment with the mineral carrier and the biomineral complex in an in vivo micronucleus assay using mouse peripheral blood erythrocytes *.

Treatment Groups **	Abbreviation	Number of Micronuclei per 1000 Erythrocytes	Mann–Whitney Criterion, U	
Control mice receiving saline solution	C	6.8 ± 1.29		
Control mice receiving colchicine, one day after (positive control)	CP	17.6 ± 1.26	U = 0, compared to C, *p*-value is 0.00604, significant	
Control mice receiving clinoptilolite	CZ	6.4 ± 1.82	U = 10, compared to C, *p*-value is 0.33724, insignificant	
Control mice receiving biomineral complex	CZL	5.0 ± 1.82	U = 3, compared to C, *p*-value is 0.03005, uncertain	
Irradiated mice (4 Gy)	I	10.8 ± 0.50	U = 0, compared to C, *p*-value is 0.00604, significant	
Irradiated mice receiving clinoptilolite	IZ	8.0 ± 0.58	U = 0, compared to I, *p*-value is 0.00604, significant	U = 5.5, compared to C, *p*-value is 0.08692, insignificant
Irradiated mice receiving biomineral complex	IZL	6.6 ± 0.82	U = 0, compared to I, *p*-value is 0.00604, significant	U = 10.5, compared to C, *p*-value is 0.37828, insignificant

* Significance level was taken as 0.01. ** Blood samples for all groups except CP were collected on the final day of the experiment.

**Table 2:** Alpha-diversity of mouse fecal samples.

Variant_Days *	OTU	Simpson	Shannon–Wiener	Evenness
C_0	2981	0.941	4.05	0.01926
CZ_0	1815	0.9356	3.892	0.02701
CZL_0	1978	0.9294	3.978	0.02699
C_6	1879	0.958	4.305	0.03943
CZ_6	2826	0.9658	4.564	0.03395
CZL_6	2306	0.9541	4.235	0.02995
C_12	2493	0.9387	4.172	0.026
CZ_12	2400	0.912	4.022	0.02326
CZL_12	2479	0.9391	3.872	0.01938
I_0	1851	0.9263	3.922	0.02728
IZ_0	1623	0.9421	3.862	0.02932
IZL_0	1657	0.9258	3.842	0.02814
I_6	3486	0.9537	4.512	0.02615
IZ_6	2224	0.9258	3.828	0.02067
IZL_6	2726	0.9537	4.434	0.0309
I_12	4434	0.989	5.584	0.06005
IZ_12	2380	0.9751	4.741	0.04811
IZL_12	2555	0.9353	4.24	0.02715

* Days since the beginning of the experiment.

## Data Availability

The raw data supporting the conclusions of this article will be made available by the authors on request.

## Supplementary Materials

The following supporting information can be downloaded at: https://www.mdpi.com/article/10.3390/ijms27114794/s1.

## Abbreviations

The following abbreviations are used in this manuscript:

RT	Radiotherapy
OTU	Operational taxonomic unit
ZIF	Zeolitic imidazolate framework
GIT	Gastrointestinal tract
NMR	Nuclear magnetic resonance
C	Control mice receiving saline solution
CP	Control mice receiving colchicine, one day after (positive control)
CZ	Control mice receiving clinoptilolite
CZL	Control mice receiving biomineral complex
I	Irradiated mice (4 Gy)
IZ	Irradiated mice receiving clinoptilolite
IZL	Irradiated mice receiving biomineral complex
