# Supplementary material for: Biomineral Complex with Probiotic and Detoxifying Properties for Recovery After Radiotherapy
Source: Int J Mol Sci. 2026 May 26;27(11):4794. doi: 10.3390/ijms27114794 (PMC13256299; doi:10.3390/ijms27114794)
Supplement: Supplementary file 1 [file ijms-27-04794-s001.zip › ijms-4317161-supplementary.pdf]

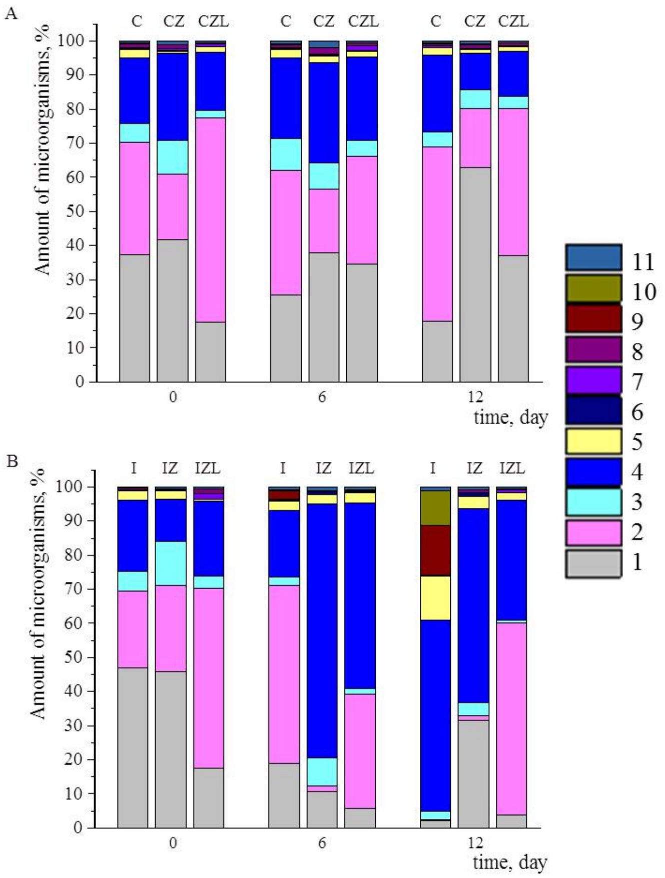

Figure S1. Dominant *Bacteroidota* phyla: A – non-irradiated mice group; B – irradiated mice (dose 4 Gy); 1 – *Muribaculaceae*; 2 – *Prevotellaceae*; 3 – *Rikenellaceae*; 4 – *Bacteroidaceae*; 5 – *Tannerellaceae*; 6 – *Barnesiellaceae*; 7 – *Incertae Sedis*; 8 – *Marinifilaceae*; 9 – *Sphingobacteriaceae*; 10 – *Flavobacteriaceae*; 11 – Other.

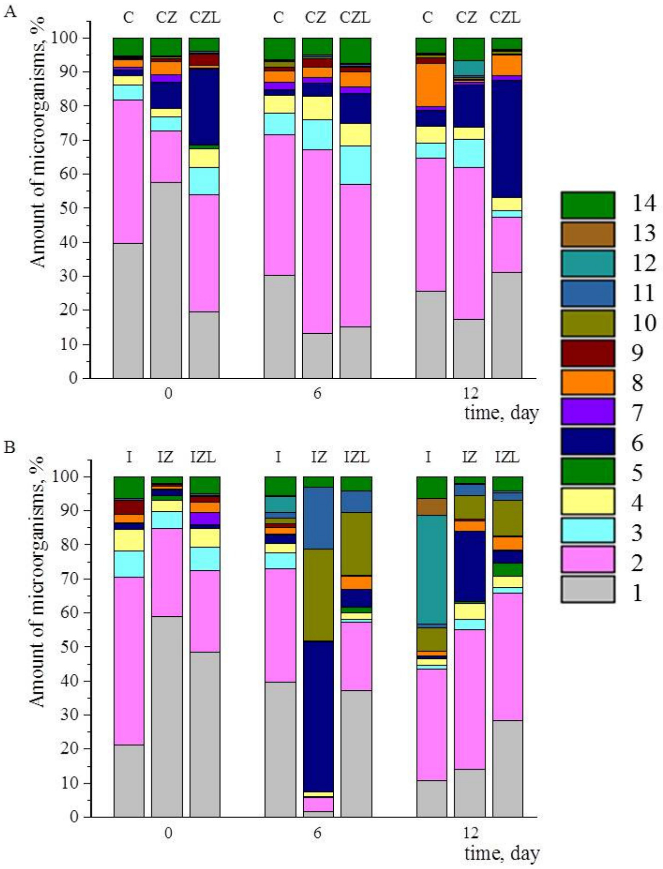

Figure S2. Dominant *Bacillota* phyla: A – non-irradiated mice group; B – irradiated mice (dose 4 Gy); 1 – *Lactobacillaceae*; 2 – *Lachnospiraceae*; 3 – *Oscillospiraceae*; 4 – *Ruminococcaceae*; 5 – *Acidaminococcaceae*; 6 – *Erysipelotrichaceae*; 7 – *Incertae Sedis*; 8 – *Erysipelatoclostridiaceae*; 9 – *Selenomonadaceae*; 10 – *Clostridiaceae*; 11 – *Peptostreptococcaceae*; 12 – *Staphylococcaceae*; 13 – *Planococcaceae*; 14 – Other.

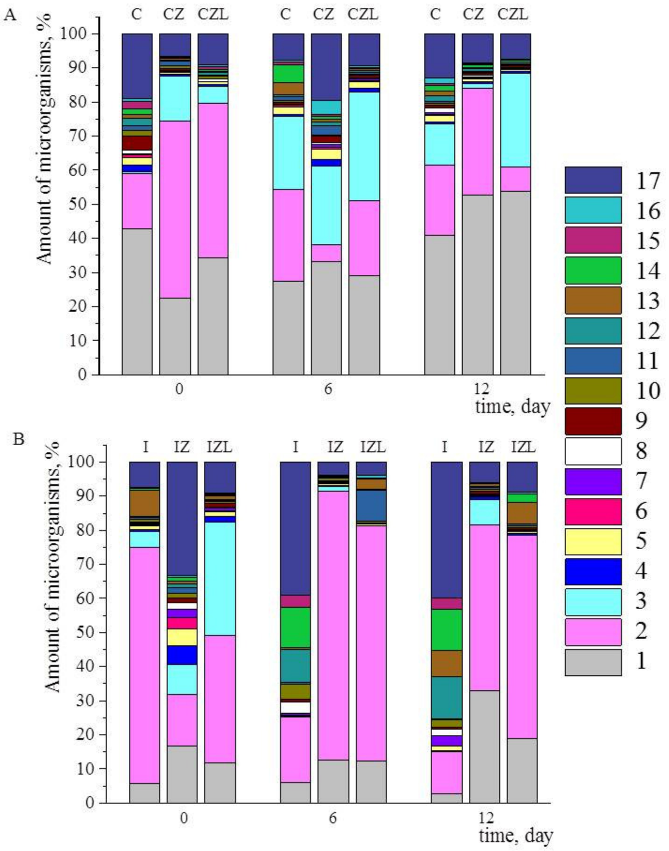

Figure S3. Dominant *Pseudomonadota* phyla: A – non-irradiated mice group; B – irradiated mice (dose 4 Gy); 1 – *Sutterellaceae*; 2 – *Enterobacteriaceae*; 3 – *Incertae Sedis*; 4 – *Acetobacteraceae*; 5 – *Caulobacteraceae*; 6 – *Xanthobacteraceae*; 7 – *Rhizobiaceae*; 8 – *Lysobacteraceae*; 9 – *Paracoccaceae*; 10 – *Erwiniaceae*; 11 – *Pasteurellaceae*; 12 – *Comamonadaceae*; 13 – *Morganellaceae*; 14 – *Moraxellaceae*; 15 – *Yersiniaceae*; 16 – *Mitochondria*; 17 – Other.
