# Supplementary material for: Biomineral Complex with Probiotic and Detoxifying Properties for Recovery After Radiotherapy
Source: Int J Mol Sci. 2026 May 26;27(11):4794. doi: 10.3390/ijms27114794 (PMC13256299; doi:10.3390/ijms27114794)
Supplement: Supplementary file 1 [file ijms-27-04794-s001.zip › Table S1.pdf]

Table S1. Predicted metabolic pathways genes copy numbers

With irradiation	I_0	I_6	I_{12}	IZ_0	IZ_6	IZ_{12}	IZL_0	IZL_6	IZL_{12}
Glycerophospholipid metabolism	4830,5	3726,84	4099,2	511	162,86	938,5	334,1	5370,65	3158,43
alpha-Linolenic acid metabolism	344,25	100,75	532,52	1	11,64	58,75	19,55	406,53	296,11
Biosynthesis of unsaturated fatty acids	130	146,64	558,17	3	14,22	58,4	13,8	29,64	10
TCA cycle	5822	3698,66	4980,95	326	219,19	1183,6	366	7456,94	2331,98
Oxidative phosphorylation	9070,75	8439,47	8870,13	736	405,03	2016,95	607,85	12225,05	4870,69
Lipopolysaccharide biosynthesis	618	791,84	918,84	19	31,06	173,5	53,9	996,34	109,72
Linoleic acid metabolism	350,75	82,29	741,17	1	4,39	58,25	5,75	388,17	1,61
Arginine biosynthesis	5413,75	4695,73	3848,5	477	221,25	1150,25	386,25	7254,37	3706,12
beta-Alanine metabolism	712,5	1485,08	2027,15	264	36,39	129,6	50,1	612,73	438,36
rginine and proline metabolism	3951,75	3770,75	4103,11	434	141,55	741,05	235,25	5640,94	3928,79
Glutathione metabolism	2449,5	2515	3347,42	191	96,96	555,8	112,7	3397,76	1033,38
Tryptophan metabolism	2089,25	1176,99	1973,63	169	72,62	352,65	100,65	1801,22	962,91
Propanoate metabolism	3593,25	4527,13	4991,46	321	194,66	905,25	280,95	5089,01	2346,93
Nitrotoluene degradation	248,75	106,81	89,92	6	4,25	1,25	8,75	46,4	0,5
Arachidonic acid metabolism	1064,25	967,39	1519,18	144	37,39	247,55	64,85	1299,23	748,83
ABC transporters	9720,5	19032,08	23178,1	2182	755,92	2072	716,1	15784,56	15987,96
Phenylalanine, tyrosine and tryptophan biosynthesis	4595	3322,04	4277,32	126	181,76	961,1	278,8	6164,96	4013,51
Butanoate metabolism	7346,5	5335,4	6765,79	565	304,58	1465,9	452,6	10524,15	4782,47
Nucleotide excision repair	1072,25	1537,85	1324,38	290	55,67	211,85	102,45	1466,67	1423,83
